# Exploring the Venom Diversity of Australian Taipans: Comparative Characterization of *Oxyuranus microlepidotus* and *Oxyuranus scutellatus*

**DOI:** 10.3390/toxins17100488

**Published:** 2025-10-01

**Authors:** Guilherme Gonelli Paz, Patrick Jack Spencer, Daniel Carvalho Pimenta, Emidio Beraldo-Neto

**Affiliations:** 1 Programa de Pós-Graduação em Ciências—Toxinologia do Instituto Butantan, São Paulo 05503-900, SP, Brazil; guilherme_gonelli@outlook.com; 2 Laboratory of Biochemistry, Instituto Butantan, São Paulo 05503-900, SP, Brazil; 3 Instituto de Pesquisas Energéticas e Nucleares, São Paulo 05508-000, SP, Brazil; pspencer@ipen.br; 4 Laboratório de Ecologia e Evolução, Instituto Butantan, São Paulo 05503-900, SP, Brazil; dcpimenta@butantan.gov.br

**Keywords:** taipan venom, proteomics, *Oxyuranus scutellatus*, *Oxyuranus microlepidotus*, Inlant Taipan, Coastal Taipan

## Abstract

The genus *Oxyuranus*, which includes some of the most venomous snakes in the world, presents a complex venom composition with potent neurotoxic and procoagulant effects. This study provides a comparative proteomic analysis of the venom of *Oxyuranus microlepidotus* (Inland Taipan) and *Oxyuranus scutellatus* (Coastal Taipan), aiming to elucidate the molecular basis underlying their distinct toxicological profiles. Using high-resolution chromatographic fractionation and LC-MS/MS, we identified a core set of nine protein families shared between both species, including phospholipases A_2_ (PLA_2_), three-finger toxins (3FTx), natriuretic peptides (NTP), nerve growth factors (NGF), and prothrombin activators (PTA). *O. microlepidotus* venom exhibited greater diversity of 3FTxs and unique protein families, such as Waprin and 5′-nucleotidases, suggesting lineage-specific functional adaptations. Quantitative analysis revealed a greater relative abundance of PLA_2_s in *O. scutellatus* (66%) compared to *O. microlepidotus* (47%), whereas 3FTXs were more prominent in *O. microlepidotus* (33% vs. 9%). These interspecific differences likely underlie the distinct clinical manifestations of envenomation and reflect evolutionary divergence in the venom composition. Our findings provide molecular insights into taipan venom complexity and highlight novel toxin candidates with potential biomedical applications in neurobiology, hemostasis, and anti-infective therapy.

## 1. Introduction

Australia is widely known for its biodiversity, especially regarding the presence of venomous and poisonous animals, including snakes [1,2,3]. Among the main snake families found on Australian territory, the *Elapidae* family stands out, whose members are known for their highly toxic venoms, with emphasis on the *Oxyuranus* genus, which includes the most venomous snakes in the world [3,4,5,6,7]. The main representatives of this genus include *O. scutellatus scutellatus* (Coastal Taipan), *O. microlepidotus* (Inland Taipan), *O. temporalis* (Central Ranges Taipan) and *O. scutellatus canni* (Papuan Taipan), the latter being found outside Australian territory [8,9,10].

Clinically, envenomation by snakes of the *Oxyuranus* genus is characterized by a set of neurotoxic and cytotoxic manifestations, including thrombocytopenia, rhabdomyolysis, acute kidney injury, and descending paralysis, which may progress to respiratory failure [7,11,12]. These clinical manifestations result from the action of various toxins present in the venom of these snakes, with taipoxin being one of the main neurotoxic components. Belonging to the phospholipase A_2_ family, this toxin induces paralysis by causing neurotransmitter depletion at motor nerve terminals [13,14,15,16,17]. Additionally, taipoxin exerts myotoxic effects through the hydrolysis of muscle fiber membranes [17].

Alongside taipoxin, taipotoxin, another toxin present in taipan venom, contributes to neurotoxic effects by blocking voltage-gated calcium channels (CaV), interfering with neurotransmission [18,19,20]. Several other toxins have been characterized in the venom of these animals, including natriuretic toxins, associated with nephrotoxic effects; prothrombin activators, responsible for coagulopathic disorders; and multiples phospholipases A_2_ [21,22,23,24]. Despite the similarity of compounds found in the venom of these taipan species, studies indicate that lethality varies between species [25,26]. Evidence suggests that *O. microlepidotus* has higher lethality compared to other taipan species and is considered the most venomous snake in the world. Nevertheless, its venom remains relatively underexplored, with few studies focused on its composition.

Several studies have investigated the venom composition of *Oxyuranus* species, employing chromatographic profiling, mass analyses, and assessment of molecular activity [8,27,28]. Nevertheless, only one study, conducted by Herrera et al. [22], has provided a comprehensive characterization of the venom of *O. scutellatus scutellatus*, in comparison with that of *O. scutellatus canni*, a subspecies found in Papua New Guinea. This study revealed a range of shared venom components between two species; however, significant quantitative differences were observed, which may account for the variation in toxicity reported between them.

Given the high specificity and potency of snake venom toxins, characterizing these molecules may also yield candidates for therapeutic development or molecular tools to study physiological processes, particularly in neurobiology and hemostasis.

Therefore, this study aims to characterize the venom of *O. microlepidotus* using proteomic approaches and compare it with the venom of *O. scutellatus*. We hypothesize that compositional differences between these species may underlie the distinct clinical outcomes of envenomation and reveal novel bioactive molecules with potential biomedical applications.

## 2. Results and Discussion

### 2.1. Venom Profile

**Figure 1:**
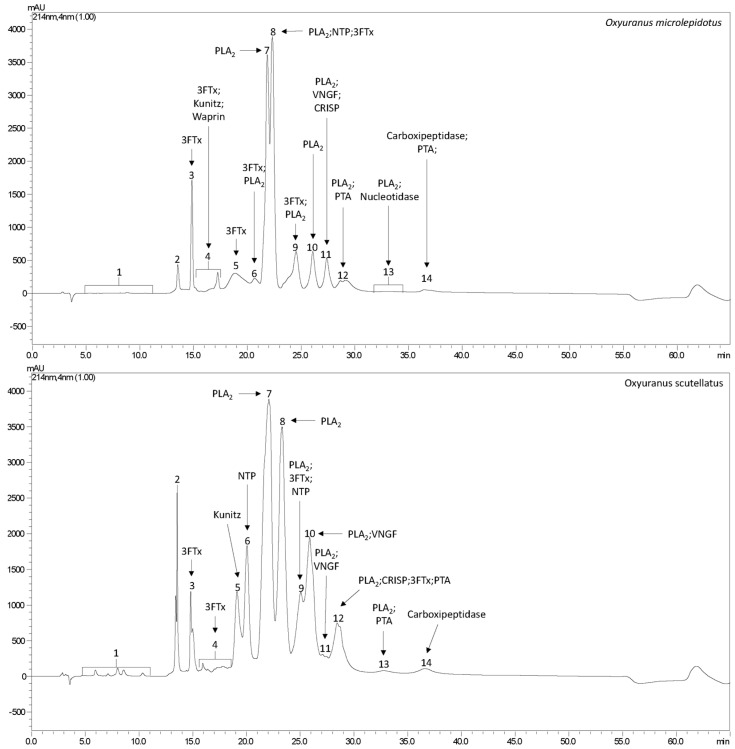
Chromatographic profile of *O. microlepidotus* and *O. scutellatus*, showing the compounds identified within the respective fractions.

**Figure 2:**
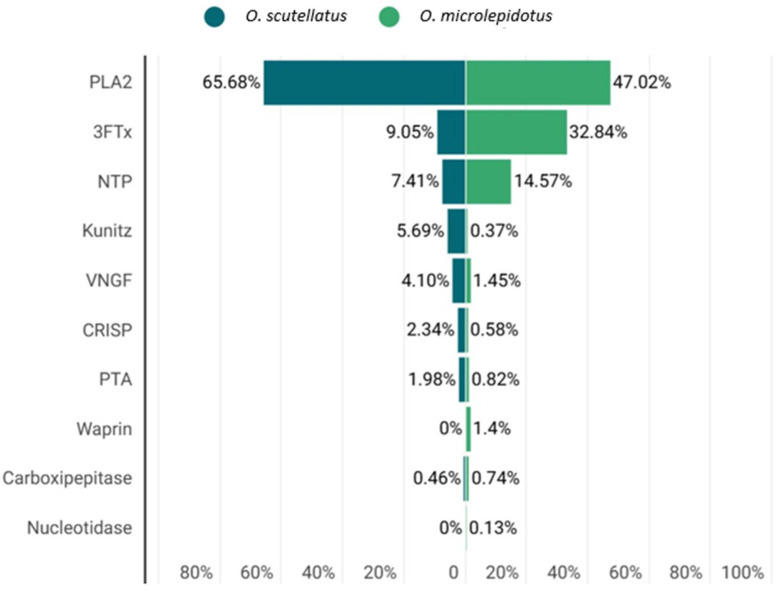
Comparative quantitative analysis of protein families identified in *O. scutellatus* and *O. microlepidotus*.

Proteomic analyses revealed the presence of nine common protein families in the venoms of *O. microlepidotus* and *O. scutellatus*, including phospholipases A_2_ (PLA_2_), three-finger toxins (3FTxs), nerve growth factors (NGFs), prothrombin activators (PTAs), natriuretic peptides (NPs), cysteine-rich secretory proteins (CRISPs), BPTI/Kunitz protease inhibitors, and carboxypeptidases (Figure 1 and Figure 2). Among these, PLA_2_ and 3FTxs stand out as the major effector molecules, being directly responsible for most of the neurotoxic and systemic effects of taipan venoms. For this reason, these two toxin families are given priority in the discussion, with emphasis on their biochemical diversity and comparative roles between the species. In addition, the proteome of *O. microlepidotus* revealed the presence of a Waprin family protein, absent in *O. scutellatus*. Waprins are structurally related to WAP (Whey Acidic Proteins) and are known for their roles in innate immunity and immunomodulation, representing a noteworthy finding that broadens the functional landscape of taipan venoms [29,30].

The Waprin identified in the venom of *O. microlepidotus* designated Omwaprin-b (B5G6G7), is a protein that shares 94% sequence identity with a previously described protein from the same species, Omwaprin-a (P83952). This protein has demonstrated antimicrobial activity through membrane disruption, without exhibiting toxicity in mice or hemolytic activity, indicating both high specificity and potent antimicrobial efficacy [31]. Furthermore, two synthetic peptides derived from Omwaprin—OMW1 and OMW2—showed even greater antimicrobial potential, effectively inhibiting *Candida albicans* biofilmformation and inducing complete lysis of *Escherichia coli* and *Staphylococcus aureus*, further highlighting the strong bioactive potential of this toxin [32]. 

**Figure 3:**
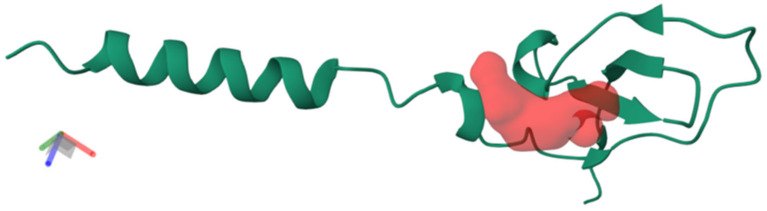
Three-dimensional structure of Omwaprin-b (B5G6G7) highlighting the identified Red Pocket. The protein backbone is represented as a ribbon diagram, while the Red Pocket is shown as a surface cavity centered at coordinates [−8.2, 7.8, 1.9]. Pocket features include a relatively small volume (103 Å^3^), high polarity, and moderate drugability score (0.49), consistent with selective recognition of polar membrane components and a potential role in antimicrobial activity.

The structural analysis of Omwaprin-b (B5G6G7) identified a single binding pocket (Figure 3) with distinct physicochemical properties. The pocket displays a moderate drugability score (0.49) and a relatively small volume (103 Å^3^), suggesting a selective interaction site for small molecules or lipid head groups. Its strongly hydrophilic character, reflected by high polarity (11.0) and negative hydrophobicity (−2.2), indicates a preference for polar or charged targets, such as phospholipid head groups or peptidoglycan components in bacterial membranes. Moreover, the high solvent-accessible surface area (1262 Å^2^) combined with a balanced polar/apolar ratio (0.91) suggests broad accessibility, which may favor initial recognition and anchoring to bacterial membranes.

These structural features are consistent with the proposed mechanism of action of cysteine-rich antimicrobial peptides, in which pocket-mediated recognition precedes membrane disruption. In the case of Omwaprin-b, the Red Pocket may function as a selective anchoring site that facilitates interaction with bacterial membrane components, ultimately leading to destabilization of bilayer integrity and cell death. This aligns with established models of antimicrobial peptide function that emphasize selective binding to bacterial versus mammalian membranes. However, since these insights are derived from computational prediction, further experimental validation is necessary to confirm the specific molecular partners and to establish the functional contribution of this pocket to Omwaprin-b’s antimicrobial activity.

In addition to the Waprin family, two proteins (A0A670YUG0; A0A2I4HXH5) belonging to the 5′-nucleotidase family were also identified in the venom of *O. microlepidotus*. The proteins share high sequence identity (96%). Although the role of these enzymes in the snake venoms is not fully understood, studies suggest that their primary function is the generation of purines, which may contribute to central nervous system depression. Additionally, they have been implicated in hemostatic modulation through the inhibition of platelet aggregation [33].

Table 1 (*O. microlepidotus*) and Table 2 (*O. scutellatus*) summarize the proteins identified in the proteomic analysis.

**Figure 4:**
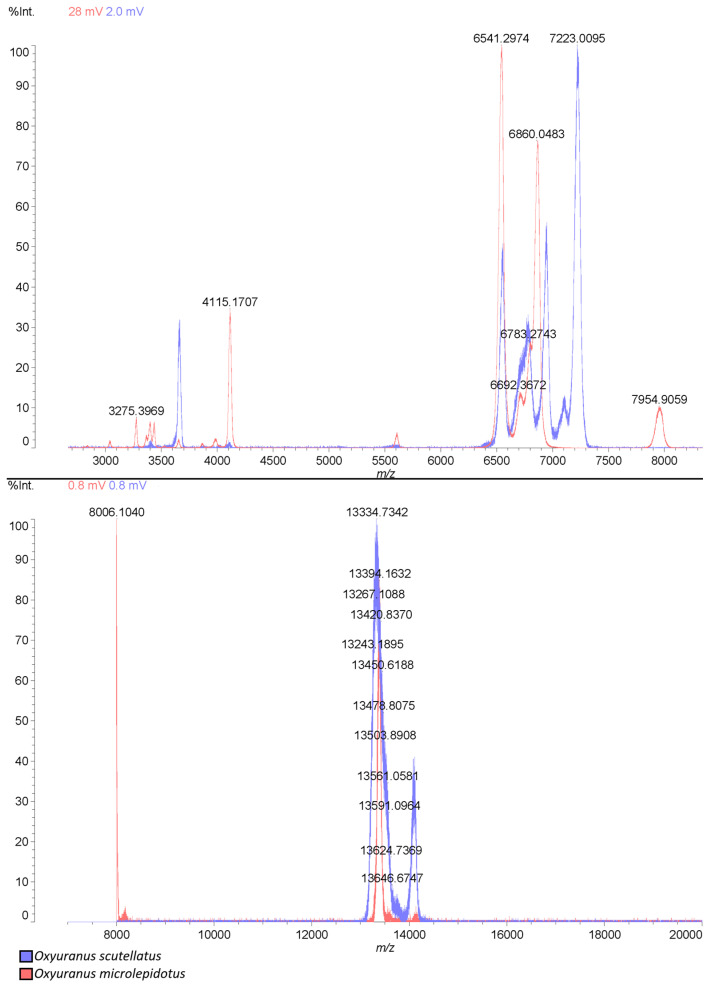
MALDI-TOF spectra from the venoms of *Oxyuranus scutellatus* (blue) and *Oxyuranus microlepidotus* (red).

To confirm the presence of the proteins identified in the proteomic analysis, the crude venom of both snake species was analyzed by MALDI-TOF mass spectrometry. As shown in Figure 4, the mass profiles of *Oxyuranus* venoms are comparable, displaying a range from 3000 *m*/*z* to 14,000 *m*/*z*. Most of the proteins identified in the venoms (Table 1 and Table 2) present theoretical masses within this range, supporting a correlation between the proteomic identifications and the experimentally observed mass spectra.

### 2.2. Phospholipases A_2_ (PLA_2_)

Both species exhibit a wide diversity of PLA_2_, as shown in Table 1 and Table 2. PLA_2_ are multifunctional enzymes present in the venoms of several snake species, primarily acting through the hydrolysis of membrane phospholipids. This enzymatic activity disrupts cellular membranes, inducing synaptic blockade through the depletion of synaptic vesicles, either by reducing endocytosis or increasing calcium permeability, which leads inhibitions of excitatory potential [13,14,15,16]—to myotoxicity, by promoting the hydrolysis of muscle fiber membranes [17].

Despite their high toxicity, PLA_2_ exhibit promising properties for the development of molecules with therapeutic potential. One study demonstrated that PLA_2_ found in the venom of *Vipera berus berus* display antimicrobial activity against Gram-positive bacteria, inhibiting their growth even when the catalytic site of the PLA_2_ is blocked, indicating that this effect is independent of its enzymatic properties [34]. Another study revealed antimalarial effects of PLA_2_ present in the venom of *Crotalus adamanteus*, inhibiting the attachment of the ookinete to the intestinal wall, also through non-enzymatic mechanisms [35]. Additionally, antitumor effects were observed in both in vitro and in vivo models using PLA_2_ isolated from *Bothrops jararacussu*, resulting in the inhibition of tumor cell proliferation [36].

**Figure 5:**
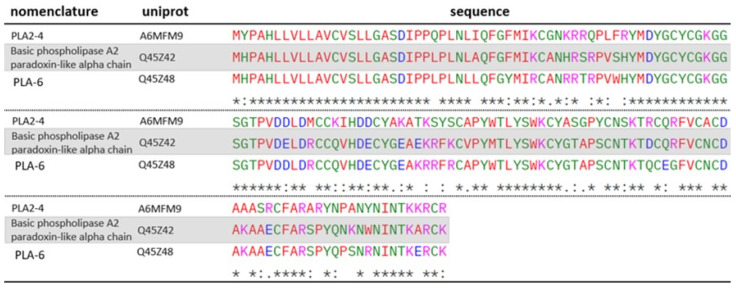
ClustalW alignment of Paradoxin alpha chain-homologous PLA_2_ identified in the venom of *O. scutellatus*: A6MFM9 and Q45Z48. Paradoxin is highlighted in gray. “*” --> fully conserved residues; “**:**” -> conservative substitution between residues; “**.**” -> semiconservative substitution between residues. Standard ClustalW color codes used for amino acid polarity/charge.

Both studies species presented Taipoxin (P00614; P0CG57; P00615; P00616) and Paradoxin (Q45Z46) in their venoms—two isoforms belonging to the PLA_2_ class—which are considered the main toxins responsible for the effects observed during envenomation by these snakes [22,23,24,25,26,27,28,29,30,31,32,33,34,35,36,37]. Taipoxin and Paradoxin are PLA_2_ of approximately 46 kDa, composed of a complex formed by three subunits: the alpha subunit, responsible for the neurotoxic action; and the beta and gamma subunits, which, although not directly active, enhance the neurotoxicity of the alpha subunit [37,38,39]. Although the alpha subunit of paradoxin was not identified in any of the venoms analyzed, two homologous proteins (A6MFM9; Q45Z48) were detected in the venom of *O. scutellatus*, with their sequence alignments presented in Figure 5. The gamma subunit of this toxin has not yet been sequenced.

Despite high sequence homology, A6MFM9 exhibits key residue substitutions relative to the Paradoxin-like alpha chain, including acidic-to-basic (E81K, D111R, E125R) and neutral-to-basic (S43R, Q73K, P131R, A143K) changes in conserved regions. These modifications increase local positive charge and raise the predicted isoelectric point (A6MFM9: pI = 8.90 vs. Paradoxin-like alpha chain: pI = 8.18), potentially affecting electrostatic interactions, structural stability, and functional properties. In contrast, Q45Z48 shows fewer substitutions, largely preserving the original charge distribution, with a predicted pI essentially identical to the reference, suggesting minimal impact on its physichochemical or functional characteristics. Independently, according to previous studies, the alpha subunit of taipoxin exhibits high enzymatic activity when isolated, with no enhancement of its activity upon complexation with the other subunit. This suggests that its enzymatic function is not directly dependent on the trimeric structure of the toxin [38]. Investigations involving paradoxin have shown that this phospholipase exerts a potent presynaptic neuromuscular blocking effect, potentially through the inhibition of potassium channels at nerve terminals, thereby suppressing neurotransmission [40].

The neurotoxic effect of the alpha subunit occurs through the alteration of the lipid composition of the axon terminal, promoting the dissociation of the Synaptophysin 1 (Syp1)-VAMP2 complex at nerve terminals. This dissociation allows Syp1 and VAMP2 to induce the formation and activation of the SNARE complex, which is essential for the docking of synaptic vesicles to the neuronal membrane, resulting in excessive neurotransmitter release and subsequent depletion [16,41,42,43,44]. In addition to their neurotoxic effects, taipoxin and paradoxin also exert myotoxic effects by promoting the degradation of the lipid layer of cell membranes [17].

**Figure 6:**
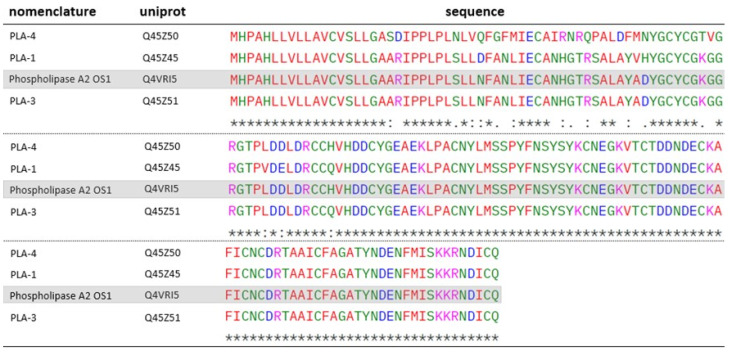
ClustalW alignment of OS1-homologous PLA_2_s identified in the venoms of *O. scutellatus*: Q45Z50, Q45Z45 and Q45Z51, and *O. microlepidotus*: Q45Z50, Q45Z45 and Q45Z51. OS1 is highlighted in gray. “*” -> fully conserved residues; “**:**” -> conservative substitution between residues; “**.**” -> semiconservative substitution between residues. Standard ClustalW color codes used for amino acid polarity/charge.

**Figure 7:**
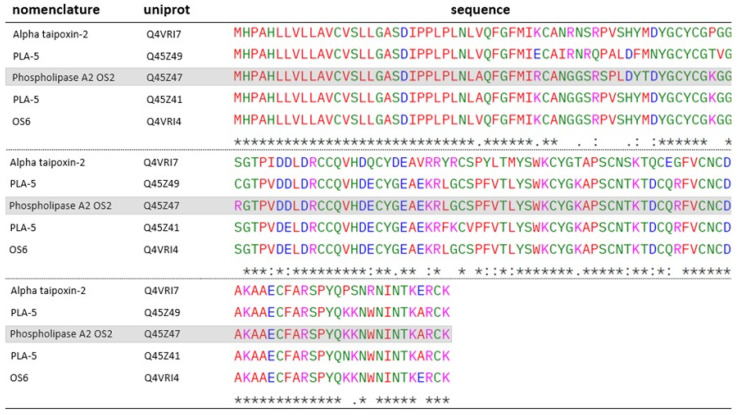
ClustalW alignment of OS2-homologous PLA_2_s identified in the venoms of *O. scutellatus*: Q4VRI7, Q45Z47 and Q4VRI4, and *O. microlepidotus*: Q4VRI7, Q45Z49 and Q45Z41. OS2 is highlighted in gray. “*” -> fully conserved residues; “**:**” -> conservative substitution between residues; “**.**” -> semiconservative substitution between residues. Standard ClustalW color codes used for amino acid polarity/charge.

In addition to these toxins, other PLA_2_ were identified in the venoms of both species, OS1 (Q4VRI5), present in both species, and OS2 (Q45Z47), identified in the venom of *O. scutellatus*, were characterized by Lambeau et al. (1990) [44]. According to the authors, both toxins bind to PLA_2_ receptors on muscle cell membranes with higher affinity than on neuronal cells. Despite targeting similar sites, only OS2 exhibited neurotoxic effects in in vivo models [45]. The action of OS2 involves the hydrolysis of phosphoglycerides present in the cell membrane in the presence of Ca^2+^, leading to tissue damage. Additionally, it can inhibit acetylcholine release at synapses, functioning as a beta-neurotoxin [21]. Other proteins identified in the venoms show homology to both OS1 (Figure 6) and OS2 (Figure 7).

Among OS1 homologs, Q45Z45 and Q45Z50 display residue substitutions that modulate their electrostatic profiles. Q45Z45 shows minor changes (N31D, D51H, H73Q) with a slight pI increase (4.94 vs. 4.79), potentially affecting local charge distribution and stability. In contrast, Q45Z50 carries multiple substitutions (T43R, R44Q, T58K, A51D, D51N), rendering it more negatively charged with a lower pI (4.56), which may influence electrostatic interactions, conformational stability, and functional properties. Q45Z51, however, closely resembles the reference (pI 4.80), suggesting minimal impact.

OS2 homologs exhibit marked residue substitutions that shift their predicted isoelectric points. Q4VRI7 (pI = 7.62) shows extensive changes, including neutral-to-basic (G41R, W137R), acidic-to-basic (D48H), basic-to-neutral (K58P, R61S, R87G, K102T, R114G, K134P, K135P), neutral-to-acidic (G80D, Q113E, A143E), and acidic-to-neutral (E77Q, E83V) substitutions, collectively increasing negative charge compared to OS2 (pI = 8.18). Q45Z49 (pI = 7.57) also harbors key substitutions—basic-to-acidic (R37E), neutral-to-basic (S43R), basic-to-neutral (R44Q, R61C, K58T), and acidic-to-neutral (D51N)—further lowering its pI. In contrast, Q4VRI4 (pI = 7.87) and Q45Z41 (pI = 8.06) share changes such as D48H and R61S, although Q4VRI4 uniquely presents a nonpolar-to-acidic substitution (A143E), rendering Q45Z41 more similar to OS2 in its electrostatic profile.

In the venom of *O. microlepidotus*, the PLA_2_ 1 (P60043) was identified, originally characterized from the venom of *Naja sagittifera* [46]. It displays a heterodimeric structure and is calcium-dependent, with Ca^2+^ ions being essential for both its structural stability and catalytic activity on phospholipids. Similarly, PLA_2_ 2 (P10116), identified in the venom of *O. scutellatus*, was initially isolated from *Laticauda colubrina*. This enzyme also exhibits Ca^2+^ dependent phospholipase activity, with a reported LD_50_ of 45 μg/kg [47].

**Figure 8:**
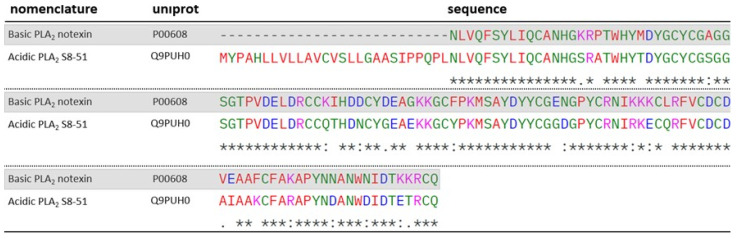
ClustalW alignment of Notexin-homologous PLA_2_ identified in the venom of *O. scutellatus*: Q9PUH0. Notexin is highlighted in gray. “*” -> fully conserved residues; “**:**” -> conservative substitution between residues; “**.**” -> semiconservative substitution between residues. Standard ClustalW color codes used for amino acid polarity/charge.

Some PLA_2_s identified in this study have not yet been functionally described in the literature; however, they share significant homology with well-characterized proteins. An example is PLA_2_ S8-51 (Q9PUH0), a protein which exhibits high sequence similarity to notexin (P00608), as shown in the alignment presented in Figure 8. Notexin, originally isolated from the venom of *Notechis scutatus scutatus*, displays typical phospholipase activity, catalyzing the hydrolysis of phospholipids in muscle or neuronal cell membranes and inhibiting acetylcholine-mediated neurotransmission. Additionally, it has been shown to exert marked nephrotoxic effects in mice following subcutaneous administration, causing glomerular and tubular damage [48,49].

Protein Q9PUH0 exhibits structural features that distinguish it from notexin. A key difference is the greater prevalence of acidic residues, arising from multiple substitutions: basic-to-neutral (K16S43, K46Q73, K116T143), basic-to-acidic (K84E111, K115E142), neutral-to-acidic (G56E83, N74D101, N107D134, N111D138), neutral-to-basic (F98K125), and acidic-to-neutral (D50N77, D53G80, E73G100, E95I122). Collectively, these changes lower its predicted isoelectric point (Q9PUH0: pI = 5.08 vs. notexin: pI = 7.39). Such alterations may influence electrostatic intramolecular interactions, potentially impacting protein stability and conformation.

PLA_2_ accounted for approximately 47% of the proteins identified in *O. microlepidotus* (Figure 2), and about 66% of the proteins in *O. scutellatus* (Figure 2), indicating a higher relative abundance of this type of toxin in *O. scutellatus* venom.

### 2.3. Three-Fingers Toxins (3FTx)

Among the identified protein families, three-finger toxins (3FTxs) represent one of the most relevant groups in taipan venoms, given their abundance, structural versatility, and central role in neurotoxicity, the 3FTX were identified in both venoms (Table 1 and Table 2). 3FTx are peptides consisting of approximately 60 to 74 amino acid residues and are found in various snake families. Their structure is composed of three beta-sheet loops connected to a central core, forming a three-dimensional conformation that resembles three fingers, hence the name [50]. These toxins are highly potent, acting through interaction with acetylcholine receptors (AChRs), where they block the action of acetylcholine, leading to inhibition of neurotransmission at the neuromuscular junction and, consequently, to paralysis [51].

As observed with PLA_2_, a greater diversity of 3FTx was identified in the venom of *O. microlepidotus*, accounting approximately 33% of the identified proteins (Figure 2), compared to about 9% of the identified protein composition in *O. scutellatus* venom (Figure 2).

**Figure 9:**
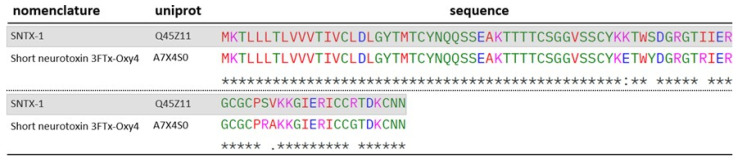
ClustalW alignment of SNTX-1-homologous 3FTx identified in the venom of *O. microlepidotus*: A7X4S0. SNTX-1 is highlighted in gray. “*” -> fully conserved residues; “**:**” -> conservative substitution between residues; “**.**” -> semiconservative substitution between residues. Standard ClustalW color codes used for amino acid polarity/charge.

The toxin SNTX-1 (Q45Z11) found in the venom of *O. scutellatus* was described by Zamudio et al. [52]. According to the authors, this toxin exhibits a high inhibitory capacity on skeletal muscle AChRs, with an IC_50_ of 2.4 ± 0.4 nM. In contrast, it does not display the same affinity for brain AChRs, suggesting a postsynaptic site of action. The authors also described SNTX-2 (P0CB06), identified in the venom of both snake species. This 3FTx exhibits a mode of action similar to that of SNTX-1. Although SNTX-1 itself was not identified in the venom of *O. microlepidotus*, a homologous protein, named 3FTx-Oxy4 (A7X4S0) was detected. The sequence alignment is shown in Figure 9.

**Figure 10:**
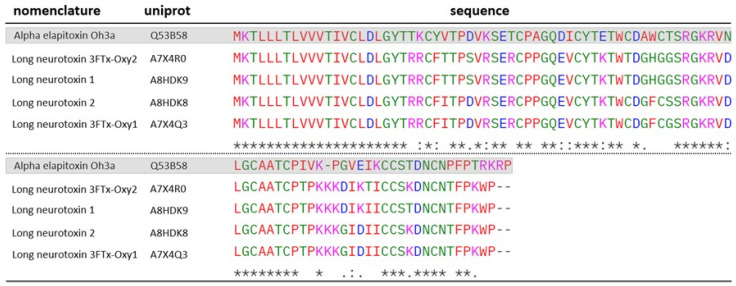
ClustalW alignment of Elapitoxin-Oh3a-homologous 3FTXs identified in the venoms of *O. microlepidotus*: A7X4R0, A8HDK8 and A7X4Q3, and *O. scutellatus*: A8HDK9. Elapitoxin-Oh3a is highlighted in gray. “*” -> fully conserved residues; “**:**” -> conservative substitution between residues; “**.**” -> semiconservative substitution between residues. Standard ClustalW color codes used for amino acid polarity/charge.

Three proteins identified in the venom of *O. microlepidotus* (A7X4R0; A8HDK8; A7X4Q3) and one protein identified in the venom of *O. scutellatus* (A8HDK9) show sequence homology to a well-characterized 3FTx, Elapitoxin-Oh3a (Q53B58). The alignment of these sequences is presented in Figure 10. This toxin was shown to induce blockade of nerve stimulation in the biventer cervicis muscle of chickens when challenged with endogenous acetylcholine and carbachol (CCh), both of which activate muscarinic and nicotinic receptors [53]. The authors also suggested a pseudo-irreversible antagonistic effect of this toxin based on a concentration-dependent, non-parallel rightward shift in the cumulative concentration-response to CCh, accompanied by a reduction in the maximal response in unstimulated chick muscle. However, this effect was not observed upon stimulation with KCl, further supporting a postsynaptic site of action for this toxin.

**Figure 11:**
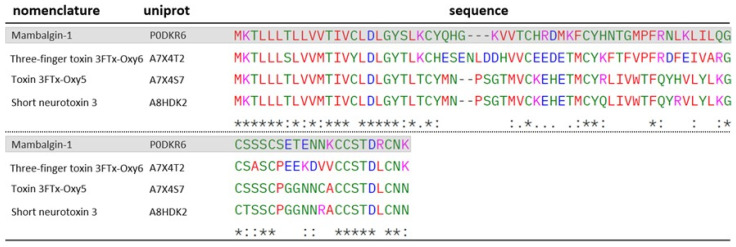
ClustalW alignment of Mambalgin-1-homologous 3FTxs identified in the venom of *O. microlepidotus*: A7X4T2, A7X4S7 and A8HDK2. Mambalgin-1 is highlighted in gray. “*”-> fully conserved residues; “**:**” -> conservative substitution between residues; “**.**” -> semiconservative substitution between residues. Standard ClustalW color codes used for amino acid polarity/charge.

3FTx possess particularly relevant characteristics for studies targeting molecular mechanisms related to pain modulation. A series of 3FTxs with inhibitory action on acid-sensing ion channels (ASICs) has been characterized from the venom of the snake *Dendroaspis polylepis*, resulting in analgesic effects comparable to those of morphine [54]. Interestingly, some proteins identified in the venom of *O. microlepidotus* show a degree of sequence homology with one of the characterized 3FTx, mambalgin-1 (P0DKR6). The alignment of these sequences is presented in Figure 11.

ASICs are expressed in both the central and peripheral nervous systems (especially in nociceptive neurons) and play a key role in signaling and propagation of pain [55]. For this reason, the inhibition of these channels by 3FTx is directly associated with the induction of analgesia.

Recent studies have also revealed that ASICs are involved in processes within the central nervous system related to the release of neurotransmitters such as GABA and glutamate, and are implicated in pathophysiological mechanisms associated with neurological disorders such as epilepsy and Alzheimer’s disease [56,57]. These findings make 3FTxs promising targets for therapeutic investigations in neuroscience.

### 2.4. Other Proteins

In addition to the dominant toxin families, several other protein groups were identified in the venoms of *O. microlepidotus* and *O. scutellatus* (Table 1 and Table 2). Nerve growth factors (NGFs) accounted for ~1.45% and ~4.10% of proteins, respectively (Figure 2). Despite limited characterization of the sequences detected (Q3HXZ0; Q3I5F4), they show homology with NGF (Supplementary Figure S1) from *Macrovipera lebetinus* (P25428), previously reported to stimulate neurite outgrowth in PC12 cells [55,58,59,60,61,62,63]. Such findings reinforce the widespread presence of NGFs in snake venoms and their potential role beyond neuronal regulation, including tissue repair and neuroinflammatory processes. Prothrombin activators (PTAs) were also detected (~0.82% in *O. microlepidotus* and ~1.98% in *O. scutellatus*), with Oscutarin-C (Q58L96; Q58L91) identified in *O. scutellatus* as a potent procoagulant toxin, and the homolog Omicarin-C (Q58L95) present in *O. microlepidotus* (Supplementary Figure S2) [22,64,65,66,67,68,69].

Natriuretic peptides (NPs) constituted one of the most abundant protein classes among this group, reaching ~14.57% in *O. microlepidotus* and ~7.41% in *O. scutellatus*. Functional differences were observed, as TNP-b (P83228) from *O. scutellatus* exhibited reduced vasorelaxant activity and failed to activate the GC-A receptor, while homologs of TNP-c (Q3SAF8; Q3SAX8) (Supplementary Figure S3) identified in both species displayed potent vasodilatory effects [70,71,72,73,74,75,76]. These peptides resemble mammalian NPs in structure and activity, suggesting their involvement in prey immobilization via hypotension and highlighting their potential as templates for cardiovascular drug discovery. CRISPs were also identified, in smaller proportions (~0.58% and ~2.34%), including homologs such as pseudecin (Supplementary Figure S4), known to inhibit cyclic nucleotide-gated channels but with limited direct toxicity [77,78,79,80,81,82,83,84]. Notably, CRISPs have been associated with anti-angiogenic and antiparasitic activities in other species, expanding their relevance beyond envenomation.

Kunitz-type inhibitors added further diversity, with taicatoxin (B7S4N9) present in both venoms (~0.37% in *O. microlepidotus* and ~5.69% in *O. scutellatus*), acting as a heterotrimeric complex that blocks cardiac CaV channels [18,19,85,86,87,88,89,90,91,92]. In addition to classical protease inhibition, Kunitz toxins have been reported to modulate ion channels and display pharmacological properties, including AVP antagonism, anti-angiogenic, and anticoagulant activities [93,94,95,96]. Finally, carboxypeptidases (~0.74% and ~2.46%) were identified in both venoms (Figure 2). Although scarcely studied in snakes, these enzymes are known to cleave peptides at the carboxy-terminal end, participate in angiotensin regulation, coagulation, and inflammatory pathways [97,98]. Taken together, these protein families highlight the functional complexity of taipan venoms, extending their biological impact beyond neurotoxicity and supporting their potential as valuable models for biomedical applications.

## 3. Conclusions

Both *Oxyuranus* venoms exhibit a shared proteomic framework dominated by PLA_2_s and 3FTxs, yet a clear divergence in toxin repertoire highlights distinct functional strategies. While *O. scutellatus* venom appears predominantly PLA_2_-driven, reinforcing its role in presynaptic neurotoxicity and myotoxicity, *O. microlepidotus* displays an expanded diversity of 3FTxs coupled with the exclusive presence of 5′-nucleotidases and Waprin, indicating a broader range of neurotoxic and cardiocirculatory mechanisms. These compositional differences suggest evolutionary pressures that may underlie the greater lethality historically attributed to *O. microlepidotus*, potentially reflecting ecological adaptation through diversification of effector molecules. The identification of Waprin, alongside several uncharacterized proteins, not only broadens the functional scope of taipan venoms but also emphasizes their unexplored pharmacological potential, positioning them as valuable resources for future studies on toxin evolution, molecular mechanisms of envenomation, and the discovery of novel therapeutic leads.

## 4. Materials and Methods

### 4.1. Venom Fractioning

The lyophilized venoms of *O. microlepidotus* and *O. scutellatus* (3 mg each), provided by Venom Supplies (Tanunda, SA) were resuspended in 0.1% Trifluoroacetic Acid (TFA) and centrifuged (10,000× *g*) for 10 min, at 4 °C. The supernatant was then fractionated by reversed-phase high-performance liquid chromatography (RP-HPLC) in a Shimadzu Prominence binary system (Shimadzu, Kyoto, Japan), coupled to a C18 analytical column (250 mm *×* 4.6 mm, 5 m). UV detection was performed (SPDM 20A, Shimadzu, λ = 214 nm) and separation was achieved by a linear gradient of 20–100% solvent B (90% acetonitrile, containing 0.1% TFA) over A (0.1% TFA) for 50 min at a constant flow of 1mL·min^−1^. The use of this strategy aimed to enhance the resolution for fractionation, and later proteomic analysis, thereby enabling more precise identification and characterization of the venom constituents.

### 4.2. Fractions Characterization

#### 4.2.1. Protein Digestion

The collected fractions (50 μL aliquots) were digested with trypsin (Sigma-Aldrich, St. Louis, MO, USA) following a standardized protocol. Initially, samples were buffed in 50 mM ammonium bicarbonate diluted in water and reduced with 5 μL of 100 mM dithiothreitol (DTT) at 60 °C for 30 min. Alkylation was performed using 5 μL of 100 mM iodoacetamide (IAA) in the dark for 45 min. Subsequently, enzymatic digestion was carried out with 240 ng of sequencing-grade trypsin at 30 °C for 16 h. Trypsin activity was quenched by the addition of 5 μL of 10% TFA, and the resulting peptide mixtures were stored at −20 °C until LC-MS/MS analysis.

#### 4.2.2. Analysis by LC-ESI-IT-TOF/MS

The samples then were analyzed with liquid chromatography mass spectrometry in an ESI-IT-TOF instrument coupled to a UPLC 20A Prominence (Shimadzu, Kyoto, Japan). Samples (50 μL aliquots) were loaded into a C18 column (Kinetex C18, 5 μm; 50 mm × 2.1 mm) and fractionated by a binary gradient employing as solvents (A) water:acetic acid (999:1) and (B) ACN:water:acetic acid (949:50:1). An elution gradient of 0–40% B was applied for 80 min at a constant flow of 0.2 mL·min^−1^ after initial isocratic elution for 5 min. The eluates were monitored using a Shimadzu SPD-M20A PDA detector before being injected into the mass spectrometer.

The interface was kept at 4.5 kV and 200 °C. The detector operated at 1.95 kV and the argon collision induced fragmentation was set at 55 ‘energy’ value. MS spectra were acquired in positive mode in the 350–1400 *m*/*z* range, and MS/MS spectra were collected in the 50–1950 *m*/*z* range.

#### 4.2.3. Analysis by MALDI-TOF/MS

The venoms were also analyzed using a matrix-assisted laser desorption ionization-time of flight (MALDI-TOF) mass spectrometer (Axima Performance, Shimadzu, Kyoto, Japan). One microliter of each sample was co-crystallized with 1 μL of α-cyano-4-hydroxycinnamic acid matrix (Sigma-Aldrich, St. Louis, MO, USA) for the low *m*/*z* range (1000~10,000) and with 1 μL of 3,5-dimethoxy-4-hydroxycinnamic acid (Sigma-Aldrich, St. Louis, MO, USA) for the high *m*/*z* range (8000~20,000). The plate containing the samples was then dried at room temperature. The mass spectrum was obtained in linear positive mode.

#### 4.2.4. Proteomic Data Processing and Data Analysis

Raw LCD Shimadzu datafiles were converted into mzML files using the LCMSolution tool and then loaded into Peaks Studio V7.0 (Bioinformatic Solutions Inc., Waterloo, ON, Canada). Data were processed according to the following parameters: MS and MS/MS error mass were 0.1 Da; methionine oxidation and carbamidomethylation as variable and fixed modification, respectively; trypsin as cleaving enzyme; maximum missed cleavages (3), maximum variable PTMs per peptide (3), and non-specific (1). Data were analyzed against the “Snakes” database, compiled in December/2024 by UNIPROT. Only peptides with a −10logP (20) and proteins with a −10logP (20), containing at least one unique peptide, were considered in the analyses. Protein alignment was performed by Clustal Omega (v. 1.2.4) (EMBL-EBI). The theoretical mass was calculated by subtracting the mass of the signal peptide using the PeptideMass software (https://web.expasy.org/peptide_mass/, accessed on 26 August 2025).

#### 4.2.5. Quantitative Analysis of Venom Proteins

The quantitative analysis of venom proteins was performed based following the approach described by Beraldo-Neto (2023) [99]: Quantification was based on the area under the chromatographic peaks, as calculated using LCMSolution software (v 1.25), and used as a proxy for protein abundance. In cases where multiple proteins co-eluted within the same retention time (i.e., within the same fraction), the relative contribution of each protein to the total peak area was estimated by normalizing the number of unique peptides identified for each protein.

### 4.3. Computational Pocket Mapping

We used the Deep Origin platform (Balto module) to perform automated binding pocket detection and characterization on the B5G6G7 (Omwaprin-b) structure. First, the input PDB file was cleaned and preprocessed (e.g., removal of water molecules, addition of missing atoms, protonation at physiological pH) through the built-in protein preparation routines. Next, the platform’s pocket-finding algorithm was invoked to scan the protein surface for putative ligand-binding cavities. Deep Origin exposes a Pocket class within its pipeline (via deeporigin.drug_discovery.Pocket) that supports operations such as from_pdb_file, from_pocket_finder_results, and get_center, enabling extraction of pocket geometry and coordinate data. client-docs.deeporigin.io.

Once pockets were detected, the system computed a panel of pocket descriptors including volume, hydrophobicity, polarity, total solvent-accessible surface area (SASA), and drugability score. These metrics are drawn from internal scoring models that integrate physical and statistical features (e.g., topology, atom types, hydrophobic patches) to estimate drug-likeness. The pocket center coordinates were derived by averaging the atomic positions within the cavity (via the get_center() method). The default docking grid box size (24 Å) was applied around the pocket center for further docking and validation procedures. After annotation, the highest-scoring pocket (“Red Pocket”) was selected for downstream mechanistic interpretation and binding modeling.

## Figures and Tables

**Table 1:** Identified proteins in the venom of *O. microlepidotus* by proteomic analysis.

Fraction	UNIPROT Code	Theoretical Mass (kDa) ^1^	−10lgP	Peptides	Unique Peptides	Protein Description	Type
3	A7X4S0	6.79	137.57	8	8	Short neurotoxin 3FTx-Oxy4 (*O. microlepidotus*)	3FTx
4	P0CB06 ^F4^	6.79	64.89	3	1	Short neurotoxin 2 (*O. scutellatus*)	3FTx
	B7S4N9 ^F5^	7	61.68	3	3	Kunitz-type protease inhibitor taicotoxin (*O. scutellatus*)	Kunitz
	B5G6G7	5.72	119.78	12	12	Omwaprin-b (*O. microlepidotus*)	Waprin
5	A7X4R0	7.91	137.90	8	1	Long neurotoxin 3FTx-Oxy2 (*O. microlepidotus*)	3FTx
6	Q45Z49	14.32	85.63	4	1	PLA-5 (*O. scutellatus*)	PLA_2_
	A8HDK8	7.98	191.13	16	1	Long neurotoxin 2 (*O. microlepidotus*)	3FTx
	A7X4Q3	7.95	188.04	16	1	Long neurotoxin 3FTx- Oxy1 (*O. microlepidotus*)	3FTx
7	Q45Z41	14.22	152.09	12	4	PLA-5 (*O. microlepidotus*)	PLA_2_
8	Q4VRI7 ^F8^	14.35	99.72	5	1	Alpha taipoxin-2 (*O. scutellatus*)	PLA_2_
	A7X4S7	6.28	102.05	4	4	Toxin 3FTx- Oxy5 (*O. microlepidotus*)	3FTx
	Q3SAF8	4.11	123.71	6	6	Natriuretic peptide OmNP-d (fragment) (*O. microlepidotus*)	NP
9	P00614 ^F9^	13.83	138.13	12	6	Basic phospholipase A2 taipoxin alpha chain (*O. scutellatus*)	PLA_2_
	A7X4T2 ^F12^	6.87	129.48	6	6	Three-finger Toxin 3FTx- Oxy6 (*O. microlepidotus*)	3FTx
	A8HDK2 ^F9^	6.34	66.99	3	3	Short neurotoxin 3 (*O. scutellatus*)	3FTx
10	Q45Z45 ^F9^	15	173.78	18	6	PLA-1 (*O. microlepidotus*)	PLA_2_
	Q45Z51^F10^	14.95	159.33	12	2	PLA-3 (*O. microlepidotus*)	PLA_2_
	P0CG57	13.31	115.25	5	1	Neutral phospholipase A2 homolog taipoxin beta chain 2 (*O. microlepidotus*)	PLA_2_
11	Q45Z46 ^F10^	13.33	184.44	22	10	Neutral phospholipase A2 paradoxin-like beta chain (*O. microlepidotus*)	PLA_2_
	P00615 ^F11^	13.23	99.21	6	1	Neutral phospholipase A2 homolog taipoxin beta chain 1 (*O. scutellatus*)	PLA_2_
	Q3HXZ0 ^F10^	13.39	116.17	5	5	Venom nerve growth factor 2 (*O. microlepidotus)*	NGF
	Q3SB07 ^F12^	23.55	83.52	3	2	Cysteine-rich venom protein pseudechetoxin-like (*O. scutellatus)*	CRISP
12	P00616 ^F12^	14.60	214.03	27	16	Acidic phospholipase A2 homolog taipoxin gamma chain (*O. scutellatus*)	PLA_2_
	Q9PUH0	13.44	53.51	2	1	Acidic phospholipase A2 S8-51 (*Austrelaps superbus*)	PLA_2_
	Q58L95	15.99	85.41	3	3	Venom prothrombin activator omicarin-C catalytic subunit (*O. microlepidotus*)	PTA
	P60043	13.21	63.09	3	1	Basic phospholipase A2 1 (Fragment) (*Naja. Sagittifera*)	PLA_2_
13	Q45Z50 ^F13^	15.21	171.55	5	2	PLA-4 (*O. scutellatus*)	PLA_2_
	Q4VRI5 ^F9^	14.10	123.21	4	1	Phospholipase A2 OS1 (*O. scutellatus*)	PLA_2_
	A0A670YUG0	48.31	74.98	3	1	5′ nucleotidase ecto (*Pseudonaja textilis*)	Nucleotidase
	A0A2I4HXH5	57.81	70.13	3	1	Snake venom 5′-nucleotidase (*Naja atra*)	Nucleotidase
14	A0A670Y1M6 ^F14^	53.81	134.99	12	6	Prolylcarboxypeptidase (*Pseudonaja textilis*)	Peptidase
	A0A8C6XE75	51.58	107.01	8	1	Prolylcarboxipeptidase (*Naja naja*)	Peptidase
	V8NPH4 ^F14^	42.76	75.53	4	2	Lysosomal Pro-X carboxypeptidase (*Ophiophagus hannah*)	Peptidase

^1^ The theoretical mass was calculated by subtracting the mass of the signal peptide using the PeptideMass online software. ^F4, F5, F8–F14^ Corresponds to the venom fraction (F) of *O. scutellatus* in which the same protein is present.

**Table 2:** Identified proteins in the venom of *O. scutellatus* by proteomic analysis.

Fraction	UNIPROT Code	Theoretical Mass (kDa) ^1^	−10lgP	Peptides	Unique Peptides	Protein Description	Type
3	Q45Z11	6.73	122.20	6	1	Short neurotoxin 1 (*O. scutellatus*)	3FTx
4	P0CB06 ^F4^	6.79	94.79	4	4	Short neurotoxin 2 (*O. scutellatus*)	3FTx
	A8HDK9	7.89	67.94	5	5	Long neurotoxin 1 *(O. scutellatus*)	3FTx
5	B7S4N9 ^F4^	7	142.30	8	7	Kunitz-type serine protease inhibitor taicotoxin (*O. scutellatus*)	Kunitz
6	P83228	3.66	82.03	3	3	Natriuretic peptide TNP-b (*O. scutellatus*)	NP
7	Q45Z47	13.33	178.33	14	2	Phospholipase A2 OS2 (*O. scutellatus*)	PLA_2_
	Q4VRI4	14.2	172.76	14	1	OS6 (*O. scutellatus*)	PLA_2_
	P10116	13.35	78.09	4	1	Basic phospholipase A2 2 (*Laticauda colubrina*)	PLA_2_
	A0A898INR6	14.71	71.72	2	1	Phospholipase A (2) (*Calliophis bivirgatus*)	PLA_2_
8	Q4VRI7 ^F8^	14.35	157.86	10	5	Alpha taipoxin-2 (*O. scutellatus*)	PLA_2_
	Q45Z48	14.59	144.13	7	2	PLA-6 (*O. scutellatus*)	PLA_2_
	A6MFM9	14.52	101.38	3	2	PLA2-4 (*Crytophis nigrescens*)	PLA_2_
	P14615	13.42	50.97	2	1	Neutral phospholipase A2 3 (*Bungarus fasciatus*)	PLA_2_
9	Q45Z45 ^F10^	15	122.02	7	1	PLA-1 (*O. microlepidotus*)	PLA_2_
	P00614 ^F9^	13.83	179.92	13	8	Basic phospholipase A2 taipoxin alpha chain (*O. scutellatus*)	PLA_2_
	Q4VRI5 ^F13^	14.1	172.67	11	2	Phospholipase A2 OS1 (*O. scutellatus*)	PLA_2_
	A6MFM9	14.52	65.31	3	2	PLA2-4 (*Crytophis nigrescens*)	PLA_2_
	A8HDK2 ^F9^	6.34	102.63	4	4	Short neurotoxin 3 (*O. scutellatus*)	3FTx
	Q3SAX8	4.11	96.42	5	4	Natriuretic peptide OsNP-d (fragment) (*O. scutellatus*)	NP
10	Q45Z46 ^F11^	13.33	125.32	8	3	Neutral phospholipase A2 paradoxin-like beta chain (*O. microlepidotus*)	PLA_2_
	Q45Z51 ^F10^	14.95	171.88	10	5	PLA-3 (*O. scutellatus*)	PLA_2_
	Q3HXZ0 ^F11^	13.39	64.42	3	3	Venom nerve growth factor 2 (*O. microlepidotus*)	NGF
11	P00615 ^F11^	13.24	170.01	16	9	Neutral phospholipase A2 homolog taipoxin beta chain 1 (*O. scutellatus*)	PLA_2_
	P0CG57	13.31	132.04	9	1	Neutral phospholipase A2 homolog taipoxin beta chain 2 (*O. scutellatus*)	PLA_2_
	Q3I5F4	13.38	99.42	4	4	Venom nerve growth factor (*O. scutellatus*)	NGF
12	P00616 ^F12^	44.72	172.56	12	9	Acidic phospholipase A2 homolog taipoxin gamma chain (*O. scutellatus*)	PLA_2_
	A7X4T2 ^F9^	6.87	83.17	2	2	Three-finger toxin 3FTx-Oxy6 (*O. microlepidotus*)	3FTx
	Q58L96	44.69	128.30	6	6	Venom prothrombin activator oscutarin-C catalytic subunit (*O. scutellatus*)	PTA
	Q3SB07 ^F11^	23.55	151.14	16	3	Cysteine-rich venom protein pseudechetoxin-like (*O. scutellatus*)	CRISP
	Q8UW11	24.54	94.05	7	1	Cystein-rich venom protein 2 (*Hydrophis hardwickii*)	CRISP
13	Q45Z50 ^F13^	15.21	135.44	5	1	PLA-4 (*O. scutellatus*)	PLA_2_
	Q58L91	157.79	200.61	34	3	Venom prothrombin activator oscutarin-C non catalytic subunit (*O. scutellatus*)	PTA
14	A0A670Y1M6 ^F14^	53.81	130.34	12	6	Prolylcarboxypeptidase (*Pseudonaja textilis*)	Peptidase
	A0A8C6XDV9	54.70	117.08	8	1	Prolylcarboxipeptidase (*Naja naja*)	Peptidase
	V8NPH4 ^F14^	42.76	78.45	4	2	Lysosomal Pro-X carboxypeptidase (*Ophiophagus hannah*)	Peptidase

^1^ The theoretical mass was calculated by subtracting the mass of the signal peptide using the PeptideMass software. ^F4, F8–F14^ Corresponds to the venom fraction (F) of *O. microlepidotus* in which the same protein is present.

## Data Availability

The proteomic data presented in this study are available on request from the corresponding author due to the confidentiality clauses reason.

## Supplementary Materials

The following supporting information can be downloaded at: https://www.mdpi.com/article/10.3390/toxins17100488/s1, Figure S1: ClustalW alignment of NGF-homologous NGF identified in the venoms of *O. microlepidotus*: Q3HXZ0, and *O. scutellatus*: Q3HXZ0 and Q3I5F4. NGF is highlighted in gray. “*****” -> fully conserved residues; “**:**” -> conservative substitution between residues; “**.**” -> semiconservative substitution between residues. Standard ClustalW color codes used for amino acid polarity/charge; Figure S2: ClustalW alignment of Oscutarin-C-homologous VPTA identified in the venom of *O. microlepidotus*: Q58L95. Oscutarin-C is highlighted in gray. “*****” -> fully conserved residues; “**:**” -> conservative substitution between residues; “**.**” -> semiconservative substitution between residues. Standard ClustalW color codes used for amino acid polarity/charge. Figure S3: ClustalW alignment of TNP-c-homologous NP identified in the venom of *O. microlepidotus*: Q3SAF8 and Q3SAX8. TNP-c is highlighted in gray. “*****” -> fully conserved residues; “**:**” -> conservative substitution between residues; “**.**” -> semiconservative substitution between residues. Standard ClustalW color codes used for amino acid polarity/charge. Figure S4: ClustalW alignment of Pseudecin-homologous CRISPs identified in the venoms of *O. microlepidotus*: Q3SB07, and *O. scutellatus*: Q3SB07 and Q8UW11. Pseudecin is highlighted in gray. “*****” -> fully conserved residues; “**:**” -> conservative substitution between residues; “**.**” -> semiconservative substitution between residues. Standard ClustalW color codes used for amino acid polarity/charge.
