# Supplementary material for: Exploring the Venom Diversity of Australian Taipans: Comparative Characterization of Oxyuranus microlepidotus and Oxyuranus scutellatus
Source: Toxins (Basel). 2025 Oct 1;17(10):488. doi: 10.3390/toxins17100488 (PMC12567702; doi:10.3390/toxins17100488)
Supplement: Supplementary file 1 [file toxins-17-00488-s001.zip › toxins-3870070-supplementary.pdf]

Supplementary Figures

nomenclature	uniprot	sequence
Venom nerve growth factor	P25428	MSMLCYTLIIAFLIGIWAAPKSEDNVSLGSPATPDLSDTSCAKTHEALKTSRNTDQHYPA
Venom nerve growth factor 2	Q3HXZ0	MSMLCYTLIIAFLIGIWAAPKSEDNVPLGSPATSDLSDTSCAQTHEGLKTSRNTDQHHPA
Venom nerve growth factor	Q3I5F4	MSMLCYTLIIVFLIGIWAAPKSEDNVPLGSPATSDLSDTSCAQTHEGLKTSRNTDQRHPA
		**********.*************** ****** ********:***.*********::**
Venom nerve growth factor	P25428	PKKAEDQEFGSAANIIVDPKLFQKRRFQSPRVLFSTQPPPLSRDEQSVEFLDNADSLNRN
Venom nerve growth factor 2	Q3HXZ0	PKKAEDQELGSAANIIVDPKLFQKRRFQSPRVLFSTQPPPLSRDEQSVEFLDNEDTLNRN
Venom nerve growth factor	Q3I5F4	PKKAEDQELGSAANIIVDPKLFQKRRFQSPRVLFSTQPPPLSRDEQSVEFLDNEDTLNRN
		********:** *:****
Venom nerve growth factor	P25428	IRAKRATHPVHNRGEFSVCDSVSVWVANKTTATDIRGNVVTVMVDVKLNNNVYRQYFFET
Venom nerve growth factor 2	Q3HXZ0	IRAKRETHPVHNLGEYSVCDSISVWVANKTKAMDIKGKPVTVMVDVNLNNHVFKQYFFET
Venom nerve growth factor	Q3I5F4	IRAKRETHPVHNLGEYSVCDSISVWVANKTEAMDIKGKPVTVMVDVNLNNHVFKQYFFET
		***** ****** **:*****:******** * **:*: *******:***:*::******
Venom nerve growth factor	P25428	KCKNPSPVSSGCRGIDAKHWNSYCTTTDTFVRALTMEGNQASWRFIRIDTACVCVISRKN
Venom nerve growth factor 2	Q3HXZ0	KCRNPNPVPSGCRGIDSGHWNSYCTTTQTFVRALTMEGNQASWRFIRIDTACVCVISRKT
Venom nerve growth factor	Q3I5F4	KCRNPNPVPSGCRGIDSGHWNSYCTTTQTFVRALTMEGNQASWRFIRIDTACVCVISRKT
		**:**.** *******: *********:*******************************.
Venom nerve growth factor	P25428	DNFG
Venom nerve growth factor 2	Q3HXZ0	ENF-
Venom nerve growth factor	Q3I5F4	ENF-
		:**

Figure S1. ClustalW alignment of NGF-homologous NGF identified in the venoms of *O. microlepidotus*: Q3HXZ0, and *O. scutellatus*: Q3HXZ0 and Q3I5F4. NGF is highlighted in gray. "*"-> fully conserved residues; "**:**" -> conservative substitution between residues; "**.**" -> semiconservative substitution between residues. Standard ClustalW color codes used for amino acid polarity/charge.

nomenclature	uniprot	sequence
Oscutarin-C catalytic subunit	Q58L96	MAPQLLLCLILTFLWSLPEAESNVFLKSKVANRFLQRTKRANSLYEEFRSGNIERECIEE
Omicarin-C catalytic subunit	Q58L95	MAPQLLLCLILTFLWSLPEAESNVFLKSKVANRFLQRTKRANSLFEEFRSGNIERECIEE
		:*************
Oscutarin-C catalytic subunit	Q58L96	RCSKEEAREVFEDDEKTETFWNVYVDGDQCSSNPCHYRGTCKDGIGSYTCTCLSGYEGKN
Omicarin-C catalytic subunit	Q58L95	RCSKEEAREVFEDDEKTETFWNVYVDGDQCSSNPCHYRGTCKDGIGSYTCTCLFGYEGKN
		*** ******
Oscutarin-C catalytic subunit	Q58L96	CERVLYKSCRVDNGNCWHFCKPVQNDIQCSCAEGYLLGEDGHSCVAGGNFSCGRNIKTRN
Omicarin-C catalytic subunit	Q58L95	CERVLYKSCRVDNGNCWHFCKPVQNDIQCSCAEGYLLGEDGHSCVAGGNFSCGRNIKTRN
		**
Oscutarin-C catalytic subunit	Q58L96	KREASLPDFVQSQNAILLKKSDNPSPDIRIVNGMDCKLGECPWQAVLVDEKEDAFCGGTI
Omicarin-C catalytic subunit	Q58L95	KREASLPDFVQSQNATLLKKSDNPSPDIRIVNGMDCKLGECPWQAVLVDEKEGVFCGGTI
		*************** ************************************..******
Oscutarin-C catalytic subunit	Q58L96	LSPIYVLTAAHCINQTKMISVVVGEINISRKNPGRLLSVDKIYVHQKFVPPKKGYEFYEK
Omicarin-C catalytic subunit	Q58L95	LSPIYVLTAAHCINQTEKISVVVGEIDKSRVETGHLLSVDKIYVHKKFVPPKKGYKFYEK
		****************: ********: ** : *:**********:*********:****
Oscutarin-C catalytic subunit	Q58L96	FDLVSYDYDIAILQMKTPIQFSENVVPACLPTADFANQVLMKQDFGIVSGFGRIFEKGPQ
Omicarin-C catalytic subunit	Q58L95	FDLVSYDYDIAIIQMKTPIQFSENVVPACLPTADFANQVLMKQDFGIISGFGRIFEKGPK
		************:**********************************:***********:
Oscutarin-C catalytic subunit	Q58L96	SKTLKVLKVPYVDRHTCMLSSESPITPTMFCAGYDTLPRDACQGDSGGPHITAYRDTHFI
Omicarin-C catalytic subunit	Q58L95	SNTLKVLKVPYVDRHTCMVSSESPITPTMFCAGYDTLPRDACQGDSGGPHITAYRDTHFI
		*:****************:***
Oscutarin-C catalytic subunit	Q58L96	TGIVSWGEGCAQTGKYGVYTKVSKFILWIKRIMRQKLPSTESSTGRL
Omicarin-C catalytic subunit	Q58L95	TGIVSWGEGCAKKGKYGIYTKVSKFILWIKRIMRQKLPSTESSTGRL
		***********:.****:*****************************

Figure S2. ClustalW alignment of Oscutarin-C-homologous VPTA identified in the venom of *O. microlepidotus*: Q58L95. Oscutarin-C is highlighted in gray. "*"-> fully conserved residues; "**:**" -> conservative

substitution between residues; “**.**” -> semiconservative substitution between residues. Standard ClustalW color codes used for amino acid polarity/charge.

nomenclature	uniprot	sequence
Natriuretic peptide TNPc	P83230	-----SDSKIGNGCFGFPLDRIGSVSGLGCNRIMQNPPKKFSGE-
Natriuretic peptide TNPd	Q3SAF8	-----SDPKIGNGCFGFPIDRIGSVSGLGCNRLVQNPPKPISGES
Natriuretic peptide OsNP-d	Q3SAX8	PAAGLSDPKIGNGCFGFPIDRIGSVSGLGCNRLVQNPPKPISGES
		** **********:*************::***** :***

Figure S3. ClustalW alignment of TNP-c-homologous NP identified in the venom of *O. microlepidotus*: Q3SAF8 and Q3SAX8. TNP-c is highlighted in gray. “*”-> fully conserved residues; “**:**” -> conservative substitution between residues; “**.**” -> semiconservative substitution between residues. Standard ClustalW color codes used for amino acid polarity/charge.

nomenclature	uniprot	sequence
Cysteine-rich venom protein 2	Q8UW11	MIAFIVLLSLAAVLQQSSGTVDFASESSNKKDYQREIVDKHNALRRSVKPTARNMLQMKW
Cystein-rich venom protein pseudecin	Q8AVA3	MIAFIVLLSLAAVLQQSSGTVDFASESSNKKNYQKEIVDKHNALRRSVKPTARNMLQMKW
Cystein-rich venom protein pseudechetoxin-like	Q3SB07	MIAFTVLLSLAAVLQQSSGTVDFASESSNKKDYRKEIVDKHNDLRRSVKPTARNMLQMKW
		**** **************************:*::******* *****************
Cysteine-rich venom protein 2	Q8UW11	NSHAAQNAKRSADRCTFAHSPEHTRTVGKFRCGENIFMSSQPFAWSGVVQAWYDEIKNFV
Cystein-rich venom protein pseudecin	Q8AVA3	NSHAAQNAKRWADRCTFAHSPPNTRTVGKLRCGENIFMSSQPFPWSGVVQAWYDEIKNFV
Cystein-rich venom protein pseudechetoxin-like	Q3SB07	NSRAAQNAKRWANRCTFAHSPPYTRTVGKLRCGENIFMSSQPFAWSGVVQAWYDEVKKFV
		**:******* *:******** ******:************* ***********:*:**
Cysteine-rich venom protein 2	Q8UW11	YGIGAKPPGSVIGHYTQIVWYKSHLLGCASAKCSSTKYLYVCQYCPAGNIRSSIATPYKS
Cystein-rich venom protein pseudecin	Q8AVA3	YGIGAKPPGSVIGHYTQVVWYKSHLIGCASAKCSSSKYLYVCQYCPAGNIRGSIATPYKS
Cystein-rich venom protein pseudechetoxin-like	Q3SB07	YGIGAKPPSSVIGHYTQVVWYKSHLLGCASAKCSSTKYLYVCQYCPAGNIIGSIATPYKS
		********.********:*******:*********:************** .********
Cysteine-rich venom protein 2	Q8UW11	GPSCGDCPSACVNGLCTNPCEYEDAYTNCNDLVKERKCQTEWIKSQCPATCFCHNKII
Cystein-rich venom protein pseudecin	Q8AVA3	GPPCADCPSACVNRLCTNPCNYNNDFSNCKSLAKKSKCQTEWIKKKCPASCFCHNKII
Cystein-rich venom protein pseudechetoxin-like	Q3SB07	GPPCGDCPSACDNGLCTNPCKHNNDFSNCKALAKKSKCQTEWIKSKCPATCFCRTEII
		** *.****** * ******:::: ::**: *.*: ********.:***:***:.:**

Figure S4. ClustalW alignment of Pseudecin-homologous CRISPs identified in the venoms of *O. microlepidotus*: Q3SB07, and *O. scutellatus*: Q3SB07 and Q8UW11. Pseudecin is highlighted in gray. “*”-> fully conserved residues; “**:**” -> conservative substitution between residues; “**.**” -> semiconservative substitution between residues. Standard ClustalW color codes used for amino acid polarity/charge.
